# NLRX1 functions as a tumor suppressor in Pan02 pancreatic cancer cells

**DOI:** 10.3389/fonc.2023.1155831

**Published:** 2023-06-05

**Authors:** Margaret A. Nagai-Singer, Holly A. Morrison, Mackenzie K. Woolls, Katerina Leedy, Khan Mohammad Imran, Juselyn D. Tupik, Irving C. Allen

**Affiliations:** ^1^ Department of Biomedical Sciences and Pathobiology, Virginia-Maryland College of Veterinary Medicine, Virginia Tech, Blacksburg, VA, United States; ^2^ Graduate Program in Translational Biology, Medicine and Health, Virginia Tech, Roanoke, VA, United States; ^3^ Department of Basic Science Education, Virginia Tech Carilion School of Medicine, Roanoke, VA, United States

**Keywords:** pattern recognition receptor, pancreatic cancer, Pan02, nod-like receptor (NLR), tumor suppressor, innate immunity

## Abstract

Pancreatic cancer is a deadly malignancy with limited treatment options. NLRX1 is a unique, understudied member of the Nod-like Receptor (NLR) family of pattern recognition receptors that regulates a variety of biological processes that are highly relevant to pancreatic cancer. The role of NLRX1 in cancer remains highly enigmatic, with some studies defining its roles as a tumor promoter, while others characterize its contributions to tumor suppression. These seemingly contradicting roles appear to be due, at least in part, to cell type and temporal mechanisms. Here, we define roles for NLRX1 in regulating critical hallmarks of pancreatic cancer using both gain-of-function and loss-of-function studies in murine Pan02 cells. Our data reveals that NLRX1 increases susceptibility to cell death, while also suppressing proliferation, migration, and reactive oxygen species production. We also show that NLRX1 protects against upregulated mitochondrial activity and limits energy production in the Pan02 cells. Transcriptomics analysis revealed that the protective phenotypes associated with NLRX1 are correlated with attenuation of NF-κB, MAPK, AKT, and inflammasome signaling. Together, these data demonstrate that NLRX1 diminishes cancer-associated biological functions in pancreatic cancer cells and establishes a role for this unique NLR in tumor suppression.

## Introduction

While pancreatic cancer is not the most prevalent cancer diagnosis, it is certainly one of the most lethal. In fact, pancreatic cancer is expected to be the third most deadly cancer in the United States in 2022 with an overall 5-year survival rate of 12% that falls to 3% if the tumor has metastasized to distant sites (1). Despite the decades of research on pancreatic cancer and its potential treatments, there has been an overall increase in death rates since 1930 (2). Therefore, pancreatic cancer patients are in need of improved treatment options that are efficacious and safe. For example, recent developments in immunotherapies have propelled the immune system into the spotlight where it is recognized for its role in tumorigenesis and disease progression. These developments tend to focus on harnessing the tumor-killing functions of the adaptive immune system. For pancreatic cancer, drugs that target the PD-1/PD-L1 interaction between T cells and tumor cells are an exciting development and are approved for a small subset of patients (3). Additionally, pancreatic cancer vaccines using radiation-treated pancreatic cancer cells and GM-CSF to stimulate systemic anti-tumor immunity are currently being tested in clinical trials (4, 5). Beyond the adaptive immune response, the innate immune system provides an often-overlooked route to activate tumor-killing immune cells and alter important signaling pathways that can dictate disease outcomes (6). Specifically, pattern recognition receptors (PRRs) constitute one aspect of innate immunity that regulates the immune response and cancer-associated pathways and are promising targets for drug development (7).

PRRs constitute an arsenal of diverse intracellular and membrane-bound receptors that recognize molecular patterns associated with pathogens (pathogen associated molecular patterns or PAMPs) and damage (damage associated molecular patterns or DAMPs). There are 5 classes of PRRs, including Toll-like receptors (TLRs) and C-type lectin receptors (CLRs) in the cell membrane and RIG-I-like receptors (RLRs), AIM2-like receptors (ALRs), and NOD-like receptors (NLRs) in the cytosol (8). Typically functioning as scaffolding proteins, PRRs facilitate the formation of various multiprotein complexes that regulate downstream pathways and often elicit an immune response to address the PAMP or DAMP.

The best characterized family of NLRs facilitate the formation of a multiprotein complex known as the “inflammasome” that generates the pro-inflammatory cytokines IL-18 and IL-1β and can initiate an inflammatory type of cell death known as pyroptosis (9–11). Inflammasomes have been implicated in many types of cancer, including in pancreatic cancer where the NLRP3 inflammasome modulates inflammation and single nucleotide polymorphisms (SNPs) in the *NLRP3* gene are common in pancreatic cancer patients (12, 13). However, a subset of NLRs do not function through inflammasome formation and instead regulate inflammation through other mechanisms. These include two NLRs, NOD1 and NOD2, that promote inflammation by recruiting RIP2 to the “NODosome” and subsequently activate the NF-κB and JNK pathways (14–16). Also included in this unique subset are three NLRs that negatively regulate inflammation, NLRX1, NLRP12, and NLRC3, which all interfere with NF-κB and interferon signaling and appear to interact with TRAF proteins in the formation of a so-called “TRAFasome” (17–22). As one of the non-inflammasome forming regulatory NLRs, NLRX1 is enigmatic and its functions in different diseases and tissues remains elusive.

NLRX1 was originally described in host-pathogen interactions where it limits mitochondrial antiviral signaling (MAVS) and inhibits the NF-κB pathway to turn off type 1 interferon (IFN1) production and limit overzealous inflammation (18, 19, 23–25). Generally speaking, NLRX1 appears to inhibit inflammation, interferon production, and mitochondrial metabolism, and promote autophagy, ROS production, and TNF-induced apoptosis (18, 24–36). However, these findings are not without controversy and appear to be highly dependent on the disease and cellular context (18, 24–36). Because the pathways and processes impacted by NLRX1 are important to tumorigenesis, NLRX1 has recently been studied in the context of different cancers withevidence suggesting it possesses either tumor promoting or tumor suppressing capabilities through mechanisms that have not been fully elucidated. Indeed, several studies have shown NLRX1 is protective against colon cancer, histiocytic sarcoma, hepatocellular carcinoma, and ER/PR+ breast cancer through signaling pathways including but not limited to NF-κB, MAPK, AKT, and TNF-induced apoptosis (28, 30–32, 37, 38). Conversely, NLRX1 has been found to augment tumor progression in an AOM-only model of colon cancer, HPV-induced head and neck squamous cell carcinoma, and ER/PR- breast cancer where it increases disease burden and promotes aggressive phenotypes (31, 32, 39, 40). The current literature suggests the role of NLRX1 is highly dependent on cellular context, including the origin, aggressiveness, and disease state of the cell.

To date, the role of NLRX1 in pancreatic cancer is undefined and unexplored. Here, we use a murine pancreatic ductal adenocarcinoma cell line (Pan02) to explore how the overexpression or partial loss of NLRX1 impacts the cancer-associated phenotypes of Pan02 cells. Using a stable lentiviral transduction method, we generated Pan02 cells that overexpress NLRX1 (Pan02^OE^) or knock down NLRX1 (Pan02^KD^) and their respective controls (Pan02^OE-CTL^ and Pan02^KD-CTL^). Our results demonstrate that NLRX1 diminishes the cancer-associated phenotypes of Pan02 cells and protects against cancer-associated biological functions through the regulation of NF-κB, MAPK, AKT, inflammasome, and immune recognition/activation signaling. These findings establish a preliminary role for NLRX1 as a tumor suppressor in pancreatic cancer.

## Materials and methods

### Cell culture and transduction

Pan02 cells were obtained from the National Cancer Institute DCTD Tumor Repository (NCI) and were cultured in RPMI 1640 (ATCC) supplemented with 10% fetal bovine serum (R&D Systems) and 1% penicillin streptomycin (Thermo Fisher Scientific). Cells were incubated at 37°C and 5% CO_2._ The Pan02 cells were transduced to overexpress murine NLRX1 (Pan02^OE^) with lentiviral ORF technology, and control lentiviral ORF particles were used to generate a control cell line for the overexpression system (Pan02^OE-CTL^) (Origene MR213673L4V and PS100093V). Likewise, additional Pan02 cells were transduced to knockdown murine NLRX1 (Pan02^KD^) with lentiviral shRNA technology, and scrambled shRNA particles were used to generate a control cell line for the knockdown system (Pan02^KD-CTL^) (Origene TL515304V and TR30021V). Four separate shRNA sequences were provided by the manufacturer for the knockdown system, but only one sequence effectively knocked down NLRX1 and therefore was used for all downstream studies. All transductions were performed according to the manufacturer’s protocols. Puromycin selection was used to select for successfully transduced cells using 1 μg/mL of puromycin (Santa Cruz Biotechnology) in complete culture media, and successful transduction was confirmed by GFP with a fluorescent microscope and western blot for NLRX1 (Abcam). Cells were authenticated with commercial Mycoplasma testing (Charles River Research Animal Diagnostic Services) and morphology checks. Cells were discarded before 30 passages.

### Western blotting

Protein was extracted from transduced Pan02 cells with a protein lysis buffer consisting of 2% SDS, 100mM Tris HCl, 100mM NaCl, 1X protease inhibitor (Thermo Fisher Scientific). Protein concentration was determined by BCA assay (Thermo Fisher Scientific) according to manufacturer’s protocols and samples were diluted to 20 μg/mL with reducing sample buffer (Thermo Fisher Scientific) and loaded into pre-cast 4 to 12% Bis-Tris Mini Protein Gels (Thermo Fisher Scientific). Proteins were transferred to a PVDF membrane in 1X TGE + 20% methanol, and blocked for 60 minutes in 5% milk in TBS + 0.1% Tween-20 (TBST). All antibodies were diluted 1:1000 in 5% BSA or 5% milk and incubated overnight at 4°C (CST and Abcam). Wash steps were performed using TBST and images were obtained with iBright imaging (Thermo Fisher Scientific) using an HRP-conjugated secondary antibody (CST) and SuperSignal West Pico or Dura Chemiluminescent Substrate (Thermo Fisher Scientific).

### Proliferation assays

Transduced Pan02 cells were seeded at 1 x 10^5^ cells per mL in a 12 well plate in complete media +/- 10 ng/mL TNF (PeproTech) and incubated overnight. Twenty-four hours later, cells were stained using NucBlue Live Cell Stain (Thermo Fisher Scientific) according to manufacturer’s protocols. Several images per well were acquired on a fluorescent microscope (Invitrogen EVOS M5000) and automated counting of DAPI+ nuclei was used to determine the cell count in each image. Additionally, an MTT assay was then performed according to manufacturer’s protocols (Abcam). Transduced Pan02 cells were seeded at 1 x 10^4^ cells per well in a 96 well plate in complete media and incubated overnight. The following day, media was replaced with experimental media containing complete media +/- 10 ng/mL TNF (PeproTech) and allowed to incubate for 24 additional hours.

### Cell death assays

Transduced Pan02 cells were seeded at 1 x 10^4^ cells per well in a 96 well plate in complete media and allowed to incubate overnight. Media was then replaced with complete media +/- 10 mM H_2_O_2_ (Fisher Chemical) and allowed to incubate for 24 hours. An LDH assay was performed according to manufacturer’s protocols (Thermo Fisher Scientific).

### Migration assays

A “scratch” or “wound healing” assay was used to measure migration. Transduced Pan02 cells were seeded at 1 x 10^5^ cells per well in a 12 well plate in complete media and incubated overnight. Once confluent, media was replaced with decreased serum media (1% FBS) and incubated overnight for cells to adjust to the decreased serum content. A 200 μL pipette tip was used to make 3 scratches per well and images of each scratch were taken immediately following the scratch induction (Invitrogen EVOS M5000). At 7 hours post-scratch, images of each scratch were taken at the same location of the initial image. Images were uploaded to Fiji-ImageJ, where the width of the scratch was measured several times per image per timepoint. The rate of migration was calculated as pixels per hour.

### Reactive oxygen species assays

Mitochondrial superoxide levels were determined using analysis of fluorescent microscopy images and fluorometer readings of MitoSOX staining (Thermo Fisher Scientific). For fluorescent images, transduced Pan02 cells were seeded at 1 x 10^5^ cells per mL in a 24 well plate and allowed to incubate overnight. The following day, media was replaced with experimental media of complete media +/- 10 ng/mL TNF (PeproTech) and incubated for 24 hours. MitoSOX staining (Thermo Fisher Scientific) was done according to manufacturer’s protocol and cells were counterstained with NucBlue Live Cell Stain (Thermo Fisher Scientific). Fiji-ImageJ was used to split the fluorescent channels, remove background, and measure mean gray value. For the fluorometer readings, transduced Pan02 cells were seeded at 1 x 10^4^ cells per well in a 96 well plate and allowed to incubate for 48 hours in complete media. Media was then replaced with experimental media of complete media +/- 100 ng/mL TNF (PeproTech), +/- 45% glucose for one hour. MitoSOX staining (Thermo Fisher Scientific) was done according to manufacturer’s protocol and cells were counterstained with NucBlue Live Cell Stain (Thermo Fisher Scientific). Fluorescence was measured using a fluorometer, and RFP (MitoSOX) fluorescence was corrected for DAPI (NucBlue) fluorescence to normalize superoxide levels to the number of cells per well.

### Metabolism assays

Transduced Pan02 cells were seeded at 1 x 10^4^ cells per well in a 96 well Seahorse XF96 cell culture microplate (Agilent) in complete media and allowed to attach for 3 hours. Media was then replaced with experimental media of complete media +/- 10 ng/mL TNF (PeproTech) and incubated for 24 hours. A Seahorse XF96 Mito Stress test (Agilent) was performed according to manufacturer’s protocols at the Virginia Tech Metabolism Core. Spare respiratory capacity, proton leak, and ATP production were calculated according to the Seahorse XF Cell Mito Stress Test calculations (41).

### Transcriptomics and gene expression

Transduced Pan02 cells were seeded at 1 x 10^5^ cells per well in a 6 well plate in complete media and incubated overnight. Media was replaced with experimental media composed of complete media +/- 100 μM H_2_O_2_ and incubated another 24 hours. RNA was collected using TRIzol and stored at -80°C until submission to Thermo Fisher (Clariom S, Applied Biosystems) for microarray-based transcriptomics analysis. The Transcriptome Analysis Console (TAC, Thermo Fisher and Applied Biosystems) was used to identify DEGs and top regulated pathways. Gene lists for relevant biological processes were acquired from GeneGlobe’s RT (2)Profiler PCR Array list (42).

## Results

### NLRX1 alters proliferation, cell death, migration, and ROS levels in Pan02 cells

**Figure 1:**
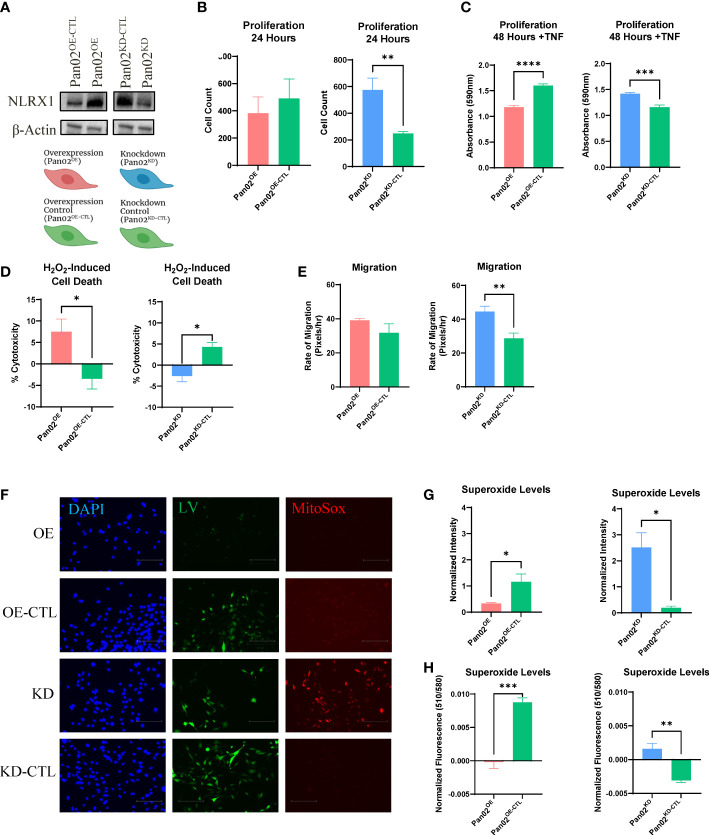
NLRX1 attenuates cancer-associated properties in Pan02 cells. **(A)** Western blot analysis of NLRX1 expression in transduced Pan02 cell lines and schematic of generated reagents and their color scheme. **(B, C).** Differences in proliferation as assessed by **(B)** automated counting and **(C)** MTT assay. **(D)** Differences in H_2_O_2_-induced cell death quantified by LDH assay. **(E)** Differences in migration as calculated by pixels per hour via scratch assay. **(F)** Representative fluorescent images of MitoSOX, an indicator for mitochondrial superoxide. DAPI shows NucBlue nuclear staining, GFP shows the GFP tag from the lentiviral construct, and RFP shows MitoSOX staining. **(G, H)**. Fluorescent intensity was measured by **(G)** Fiji-ImageJ and **(H)** a fluorometer. Data shown are representative of 1 independent experiment for all assays. n = 3-14 replicates per cell line. All quantification data were analyzed using a two-way unpaired T test and shown as mean ± SE. *p ≤ 0.05, **p ≤ 0.01, ***p ≤ 0.001, ****p ≤ 0.0001.

To elucidate what general functions NLRX1 performs when it is expressed in pancreatic cancer cells, we generated Pan02 cells to either overexpress or knockdown NLRX1, as well as their respective controls (Pan02^OE^, Pan02^OE-CTL^, Pan02^KD^, Pan02^KD-CTL^; **Figure 1A**). Importantly, due to differences between the ORF technology used for the overexpression system and the shRNA technology used for the knockdown system, we do not directly compare Pan02^OE^ to Pan02^KD^ cells. Instead, we compare them to their respective control cell lines that were generated with the same respective technology (Pan02^OE^ versus Pan02^OE-CTL^, and Pan02^KD^ versus Pan02^KD-CTL^). We then performed experiments on these four transduced Pan02 cell lines to assess common characteristics of cancer cells, including proliferation, cell death, migration, and ROS levels. To assess proliferation, we used automated microscopy counting at 24 hours and an MTT assay at 48 hours. Our Pan02 cell lines were assessed for proliferation under normal conditions or following stimulation with TNF as previously described (31, 32). At 24 hours, we found that in unstimulated conditions, the knockdown of NLRX1 in Pan02^KD^ cells more than doubled proliferation compared to their controls (**Figure 1B**). However, overexpression of NLRX1 did not impact proliferation under unstimulated conditions (**Figure 1B**). Twenty-four hours following stimulation with TNF, we observed an increase in proliferation in the Pan02^KD^ cells that was approximately 3 times greater than levels observed in the Pan02^KD-CTL^ cells (**Supplemental Figure S1A**). It should be noted that this increase was highly variable and did not achieve statistical significance, and no significant differences were observed for the overexpression cell lines at the 24-hour time point either (**Supplemental Figure S1A**). At 48 hours, we observed no differences in unstimulated conditions (**Supplemental Figure S1B**). However, following TNF stimulation at the 48-hour time point, the overexpression of NLRX1 in Pan02^OE^ cells hindered proliferation, while the knockdown of NLRX1 promoted proliferation (**Figure 1C**). Together, these data demonstrate that the partial loss of NLRX1 increases Pan02 cell proliferation under both stimulated and unstimulated conditions, and this effect on proliferation can be partially reversed by up-regulation of NLRX1. Cell death and proliferation are intricately linked in cancer cell biology. Thus, we next challenged Pan02 cells with H_2_O_2_ to induce cell death and measured cytotoxicity using an LDH assay. Tumor cells are often able to evade cell death and thus decreased Pan02 cytotoxicity would indicate a selective tumor cell advantage (43). Following the challenge, we observed significantly increased cytotoxicity in the Pan02^OE^ cells compared to the Pan02^OE-CTL^ cells (**Figure 1D**). Conversely, Pan02^KD^ cells demonstrated less cytotoxicity and therefore decreased susceptibility to H_2_O_2_-induced cell death compared to Pan02^KD-CTL^ cells (**Figure 1D**). Together, these data are consistent with the proliferation findings and demonstrate that NLRX1 promotes Pan02 cell death.

Metastasis is a major driver of cancer-associated mortality and therefore is a crucial element in reducing cancer deaths (44). As a proxy for potential metastatic ability, we next measured how NLRX1 impacts migratory capacity using a wound healing assay and calculating the rate of migration of the tumor cells. No significant differences were observed between Pan02^OE^ and Pan02^OE-CTL^ cells, but Pan02^KD^ cells demonstrated a 55% increase in migration compared to Pan02^KD-CTL^ cells (**Figure 1E**). This suggests the loss of NLRX1 in Pan02 cells promotes the migratory capabilities of the tumor cell and would indicate a potential for increased metastasis when NLRX1 expression in the tumor is decreased.

Lastly, to measure mitochondrial ROS levels that are typically upregulated in tumor cells, we used MitoSOX to stain mitochondrial superoxide and measured staining intensity with both Fiji-ImageJ and a fluorometer (45). As a positive control, cells were stimulated with glucose to induce mitochondrial ROS production and indicated no differences in maximal ROS levels (**Supplemental Figure S1C**). Under normal culture conditions, Pan02^OE^ cells had less superoxide levels than Pan02^OE-CTL^ cells in both the Fiji-ImageJ and fluorometer measurements (**Figures 1F–H**). Conversely, Pan02^KD^ cells had more superoxide levels than Pan02^KD-CTL^ cells in both the Fiji-ImageJ (**Figure 1G**) and fluorometer measurements (**Figure 1H**), and is clearly visible in the fluorescent images (**Figure 1F**). We observed similar trends in cells that were stimulated with TNF, although no statistical significance was found in the knockdown system under TNF conditions (**Supplemental Figure S1D**). Together, these data suggest that NLRX1 expressed by Pan02 cells is tumor suppressive through limiting proliferation, mitochondrial ROS levels, and migration, while also increasing cell death.

### NLRX1 protects against unregulated mitochondrial activity and limits energy production in Pan02 cells

**Figure 2:**
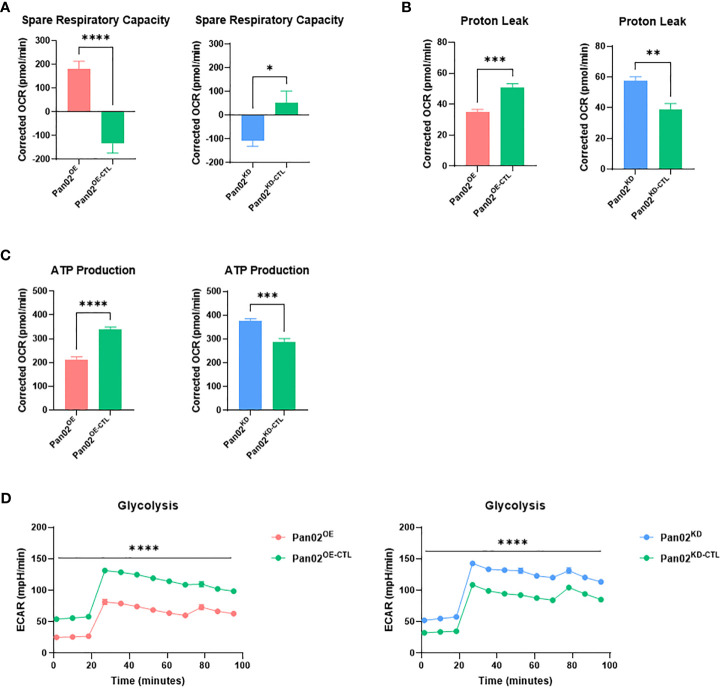
NLRX1 limits mitochondrial dysfunction and cellular energy production. **(A–D)**. From the Seahorse XF Cell Mito Stress kit, we observed differences in **(A)** spare respiratory capacity, **(B)** proton leak, **(C)** ATP production, and **(D)** glycolysis. n = 7 replicates per cell line. Data shown are representative of 1 independent experiment for all assays. All data were analyzed using a two-way unpaired T test and shown as mean ± SE. *p ≤ 0.05, **p ≤ 0.01, ***p ≤ 0.001, ****p ≤ 0.0001.

To connect the phenotypes observed in the previous section, we next sought to understand how NLRX1 impacts mitochondrial function and metabolism in Pan02 cells. Using the Seahorse XF Cell Mito Stress test, we revealed a strong role for NLRX1 in mitochondrial function. Spare respiratory capacity is an indicator of a cell’s mitochondria to perform adequately under stress conditions (46). Pan02^OE^ cells demonstrated an improved spare respiratory capacity compared to Pan02^OE-CTL^ cells, indicating that the overexpression of NLRX1 allows Pan02 cells to maintain adequate mitochondrial function when under stress (**Figure 2A**). Conversely, Pan02^KD^ cells demonstrated a decrease in spare respiratory capacity compared to Pan02^KD-CTL^ cells, indicating that the loss of NLRX1 causes Pan02 cells to poorly adapt to stressful conditions and could result in distorted mitochondria and mitochondrial dysfunction (**Figure 2A**). Indeed, a decrease in spare respiratory capacity is a common hallmark of cancer cells caused by their metabolic weakness and having “exhausted” mitochondria (46). Additionally, proton leak is typically upregulated in cancer cells as a result of their unregulated growth and mitochondrial damage (45). Consistent with that characteristic and the observed phenotypes thus far, Pan02^OE^ cells had a 33% reduction in proton leak compared to Pan02^OE-CTL^ cells while Pan02^KD^ cells had a 46% increase in proton leak compared to Pan02^KD-CTL^ cells (**Figure 2B**). These data suggest NLRX1 in Pan02 cells improves the overall health, function, and regulation of mitochondria. Conversely, the loss of NLRX1 in Pan02 cells contributes to mitochondrial dysfunction that is consistent with tumor-associated characteristics. Additionally, Pan02^OE^ cells produced 37% less ATP (**Figure 2C**) and performed less glycolysis (**Figure 2D**) than Pan02^OE-CTL^ cells, while Pan02^KD^ cells produced 31% more ATP (**Figure 2C**) and performed more glycolysis (**Figure 2D**) than Pan02^KD-CTL^ cells. This suggests that NLRX1 aids in maintaining regulation of cellular energy production in Pan02 cells. The trends observed in these data were also observed after cells were stimulated with TNF (**Supplemental Figures S2A–D**). Together, these data indicate a tumor suppressive role for NLRX1 in Pan02 cells where it protects against unregulated mitochondrial activity and limits the energy available to the cell.

### NLRX1 impacts many pathways associated with cancer and immune system regulation

**Figure 3:**
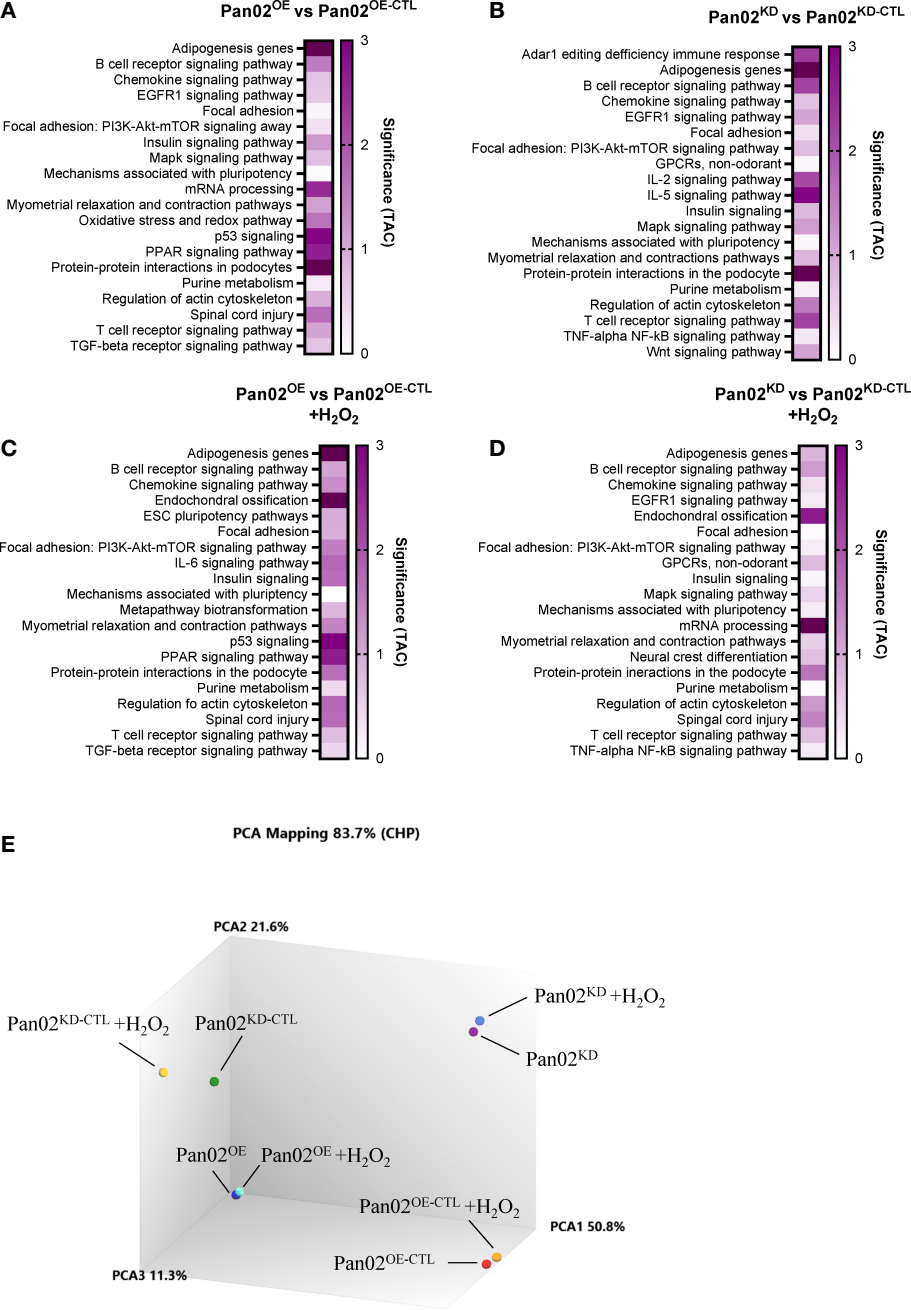
NLRX1 expression significantly modulates gene expression related to several biological functions in Pan02 cells, regardless of extracellular stress. **(A–D)**. Transcriptomics analysis of our transduced Pan02 cell lines revealed the top 20 pathways between **(A)** Pan02^OE^ and Pan02^OE-CTL^ cells in normal conditions, **(B)** Pan02^KD^ and Pan02^KD-CTL^ cells in normal conditions, **(C)** Pan02^OE^ and Pan02^OE-CTL^ cells in H_2_O_2_ conditions, and **(D)** Pan02^KD^ and Pan02^KD-CTL^ cells in H_2_O_2_ conditions. Top pathways were determined by the number of DEGs and are listed alphabetically with their significance determined in TAC. **(E)** Principal Component Analysis (PCA) mapping shows the clustering patterns of each of the 8 sample groups.

Considering the impact NLRX1 demonstrated on several cancer-associated phenotypes in our Pan02 cells, we next collected RNA from Pan02^OE^, Pan02^OE-CTL^, Pan02^KD^, and Pan02^KD-CTL^ cells for transcriptomics analysis (ClariomS). Cells were collected under normal conditions, or due to the implication of ROS levels, stress responses, and cell death in the data above, following a challenge with a low dose of H_2_O_2_. The top 50 up- and down-regulated genes under normal conditions (**Supplemental Figure S3**) and H_2_O_2_ conditions (**Supplemental Figure S4**) are listed in **Supplemental Data**. Using the Transcriptomics Analysis Console (TAC), we identified the top 20 pathways impacted by NLRX1 expression in the Pan02 cells according to the number of differentially-expressed genes (DEGs) in unstimulated or stimulated conditions (**Figures 3A–D**). Overall, there is significant overlap in the top 20 pathways between the four comparisons, which suggests that many of the pathways identified are impacted by both the loss and gain of NLRX1 expression (**Figures 3A–D**). Interestingly, many of the pathways identified here are consistent with pathways identified in previous studies of NLRX1, including PI3K-AKT, MAPK, EGFR, NF-κB, and IL-6 signaling, and these pathways are all important to the initiation and progression of pancreatic cancer (28, 30, 37, 38, 47–51). Additionally, the importance of several cytokines, B cell receptor, T cell receptor, and NF-κB signaling indicates NLRX1 is a regulator of many aspects of immune system function in this cell line. Because pancreatic cancer is typically a highly immunosuppressive tumor type and NLRX1 is best characterized for its roles in modulating immune system function, immunomodulation by NLRX1 is certainly of interest in this model (52). A Principal Component Analysis (PCA) plot revealed clustering of each Pan02 cell line together, indicating that the differences in NLRX1 expression between each transduced Pan02 cell lines is a significant driver of differences in the transcriptome (**Figure 3E**). Additionally, the presence/absence of the H_2_O_2_ challenge did not appear to significantly alter the transcriptome within each Pan02 cell line (**Figure 3E**). Together, our data identify multiple signaling pathways impacted by alterations in NLRX1 expression associated with pancreatic cancer.

### NLRX1 inhibits inflammation, immune evasion, and cancer-associated gene expression signatures

**Figure 4:**
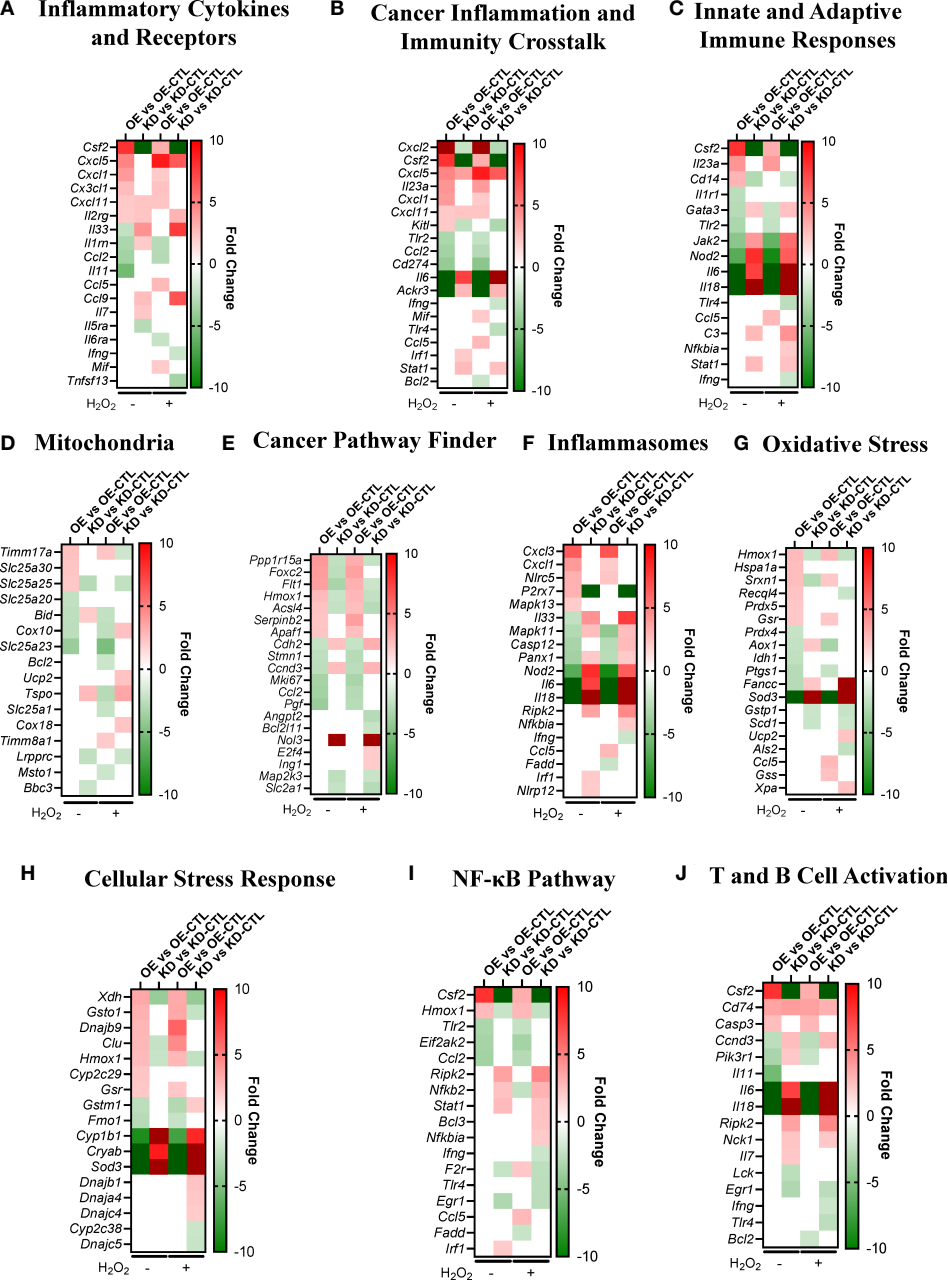
NLRX1 expression impacts signaling pathways associated with inflammation, cancer, mitochondria function, and oxidative stress in Pan02 cells. **(A–J)**. Fold change of DEGs between Pan02^OE^ and Pan02^OE-CTL^ cells in normal conditions, Pan02^KD^ and Pan02^KD-CTL^ cells in normal conditions, Pan02^OE^ and Pan02^OE-CTL^ cells in H_2_O_2_ conditions, and Pan02^KD^ and Pan02^KD-CTL^ cells in H_2_O_2_ conditions in biological processes relevant to observed *in vitro* phenotypes and top pathways. Gene lists were pulled from Qiagen/GeneGlobe.

To elaborate on the transcriptomics data, we identified the DEGs between all four comparisons in several biological processes that are important to the *in vitro* phenotypes and top pathways we identified. We identified DEGs related to inflammatory cytokines and receptors (**Figure 4A**), cancer inflammation and immunity crosstalk (**Figure 4B**), innate and adaptive immune responses (**Figure 4C**), mitochondria (**Figure 4D**), cancer pathways, (**Figure 4E**), inflammasomes (**Figure 4F**), oxidative stress (**Figure 4G**), cellular stress response (**Figure 4H**), the NF- κB pathway (**Figure 4I**), and T and B cell activation (**Figure 4J**). Genes related to these processes were pulled from the gene lists available from GeneGlobe (Qiagen). Within these biological processes, the most upregulated genes in Pan02^OE^ cells include *Csf2*, *Cxcl5*, *Cxcl2*, *Il23a*, *Timm17a*, *Slc25a30*, *Ppp1r15a*, *Foxc2*, *Cxcl3*, *Cxcl1*, *Hmox1*, *Hspa1a*, *Xdh*, *Gsto1*, and *CD74*, and the most downregulated genes in Pan02^OE^ cells include *Il11*, *Ccl2*, *Ackr3*, *Il6*, *Il18*, *Slc25a23*, *Cox10*, *Pgf*, *Sod3*, *Fancc*, *Nod2*, and *Cryab*. As we would expect, many of the most upregulated genes in Pan02^OE^ cells are also downregulated in Pan02^KD^ cells and many of the most downregulated genes in Pan02^OE^ cells are also upregulated in Pan02^KD^ cells (**Figures 4A–J**). Again, this indicates the partial loss of NLRX1 and the overexpression of NLRX1 have strong and diametric effects on Pan02 cells at the gene expression level and supports the opposing effects of the gain- or loss- of -function studies *in vitro*. The gene expression signatures here suggest a strong role for NLRX1 in limiting inflammation, including through NF-κB signaling and inflammasomes, inducing an anti-tumor immune microenvironment, and protecting against damaging cellular stress signals.

## Discussion

Here, we have established through *in vitro* assays and transcriptomics analysis that NLRX1 in murine pancreatic tumor cells is protective against cancer-associated phenotypes and likewise that the loss of NLRX1 augments cancer-associated phenotypes. Specifically, we demonstrate that the loss of NLRX1 increases proliferation, decreases cell death, promotes migration, sustains higher levels of mitochondrial ROS, contributes to mitochondrial dysfunction, and promotes unregulated energy production. As we would expect, many of these phenotypes are reversed when NLRX1 is overexpressed. Through transcriptomics analysis, we identified significant pathways regulated by NLRX1 expression and the DEGs in biological processes related to those pathways. Together, these data indicate a significant role for NLRX1 in many pathways and processes important to pancreatic cancer.

Our findings here are consistent with other studies evaluating NLRX1 function. However, NLRX1 remains an enigmatic pattern recognition receptor, in part due to the often-conflicting findings between studies. For example, in other cell lines with altered expression of NLRX1, many of the same *in vitro* phenotypes have been observed. Consistent with our studies in Pan02 cells, NLRX1 also increased cell death and decreased migration *in vitro* in two hepatocellular carcinoma cell lines (38). Likewise, the overexpression of NLRX1 in HEK293 (human embryonic kidney cells), MCF-7 (human ER/PR+ breast tumor cells), and HeLa (human cervical carcinoma cells) cell lines increased cell death and decreased ATP production (31, 32). In MCF-7 cells, the overexpression of NLRX1 also reduced clonogenicity and migration, both of which are consistent with our findings (31). However, NLRX1 overexpression in HEK293, MCF-7, and HeLa cells resulted in higher ROS levels, which is not consistent with our data (31). In another cell line, MDA-MB-231 (human ER/PR- breast tumor cells), the knockdown of NLRX1 was consistent with our results regarding ROS levels (32). However, the knockdown of NLRX1 in MDA-MB-231 cells resulted in decreased ATP, decreased proliferation, and decreased migration, all of which are inconsistent with the current study (32). While differences between cell lines can account for some differences, the current research landscape suggests that the function of NLRX1 is dependent on several factors and likely has complex spatial, temporal, and cell/tissue-specific roles (35, 36). Indeed, the expression of NLRX1 in various human neoplasms compared to healthy tissue can range from almost 3-fold increased to almost 9-fold decreased based on the type of cancer (30). Even within a specific type of cancer, such as breast cancer and hepatocellular carcinoma, the expression of NLRX1 can vary based on the subtype and aggressiveness of the specific tumor or cell line (31, 32, 38). A previous study identified fragment 556–974 of the human NLRX1 protein as being responsible for the protective phenotypes in hepatocellular carcinoma models (38), but further work is needed to elucidate how NLRX1 is able to function differently in different models and scenarios.

While the phenotypic impacts of NLRX1 expression *in vitro* offer some conflicting data throughout the literature, the biological pathways on which NLRX1 converges between several different models are consistent. Specifically, the protective roles of NLRX1 in various tumor models seem to converge on NF-κB and AKT signaling. NLRX1 has been linked with negative regulation of NF-κB signaling and subsequent protection against tumorigenesis and disease burden in gastric cancer cells challenged with *Helicobacter pylori*, intestinal organoid models of colonic tumorigenesis, AOM/DSS-induced models of colitis-associated cancer and sporadic colon cancer models in *Apc* ^min/+^ mice, urethane-induced histiocytic carcinoma, and human gastric cancer samples (28, 30, 37, 48, 49). In several of these same models and a model of hepatocellular carcinoma, NLRX1 also limits AKT signaling to protect against disease (28, 30, 38). The studies in colitis-associated cancer and sporadic colon cancer models also revealed that NLRX1 attenuates MAPK, STAT3, and IL-6 signaling pathways (37). Likewise, our transcriptomics analysis revealed an important role for NLRX1 in pancreatic cancer cells through NF-κB, AKT, MAPK, and IL-6 signaling and highlights these pathways as likely mechanisms by which NLRX1 asserts its protective qualities in Pan02 cells.

In models where NLRX1 appears to be problematic and/or contributes to more severe disease outcomes, the mechanisms center on mitochondrial function. Specifically in cancers influenced by viral infections, the ability of NLRX1 to inhibit mitochondrial interferon signaling suggests this function of NLRX1 can be detrimental to the host. Suppressing IFN-β in a model Kaposi’s sarcoma-associated herpesvirus (KSHV) suggested that NLRX1 facilitates KSHV replication and reactivation (53). Additionally, in a model of HPV+ head and neck squamous cell carcinoma (HNSCC), NLRX1 interacts with and degrades STING to decrease IFN-I production and therefore limits tumor control (39). The ability for NLRX1 to inhibit MAVS and the subsequent IFN-I signaling has also been implicated in persistent Hepatitis C (HCV) infections, which can increase the risk for many types of cancer (26, 54). Our current study did not indicate a significant role of NLRX1 in mitochondrial interferon signaling in Pan02 cells, which is not surprising as pancreatic cancer is not typically driven by viral infections. However, our results do indicate a strong association of NLRX1 with the mitochondria through metabolic and OXPHOS-related pathways and phenotypes, which have been previously reported in other models, albeit with the several inconsistent functions as discussed above (31, 32). In ER/PR- human breast cancer, which also is not driven by viral infections, NLRX1 enhanced aggressive *in vitro* cancer-associated phenotypes through augmenting mitochondrial respiration and reducing mitophagy and lysosomal formation and function through mitochondria-lysosomal crosstalk (32).

Our metabolism assays revealed that NLRX1 in Pan02 cells increases spare respiratory capacity, which indicates NLRX1 equips the cells with healthy mitochondria that are able to adapt to stressful conditions. We also discovered that NLRX1 decreases proton leak. However, because proton leak typically reduces mitochondrial superoxide production, we would expect to see a subsequent increase in superoxide levels in Pan02^OE^ cells. Conversely, we see a reduction in superoxide levels in Pan02^OE^ cells, which indicates the differences in superoxide levels are not due to proton leak but instead are likely due to the decreased basal respiration in Pan02^OE^ cells (Data not shown) (55). Interestingly, superoxide dismutase 3 (*Sod3)* was one of the most downregulated genes in Pan02^OE^ cells and also substantially upregulated in Pan02^KD^ cells. Because SOD3 reduces superoxide, this gene expression pattern might appear counterintuitive to our superoxide data. However, we believe the role of *Sod3* in Pan02 cells is primarily responsible for promoting pro-growth AKT and MAPK signaling in Pan02^KD^ cells and similarly limiting these pathways in Pan02^OE^ cells (56). Additionally, the decrease in ATP production and glycolysis also indicates that NLRX1 prevents unregulated energetics in Pan02 cells and thereby keeps their proliferation in check.

Additionally, many aspects of immune regulation were implicated in our analysis, including inflammation, chemokines, B cell and T cell signaling, NF-κB pathway, and TGF-β signaling. As we would expect based on the prior defined functions of NLRX1 in the suppression of inflammation, the pro-inflammatory cytokines *Il6* and *Il18* were significantly downregulated in the presence of excess NLRX1 and upregulated following the partial loss of NLRX1. Aberrant IL-6 and IL-18 levels both create a tumor microenvironment that is favorable for the tumor cells by promoting survival and establishing an immunosuppressive environment, suggesting that NLRX1 may protect against pancreatic tumors through regulating inflammation and inflammatory cytokines (57). The regulation of IL-18 and many other inflammasome-related genes also indicates that NLRX1 suppresses inflammasome function in Pan02 cells, albeit this is likely through the suppression of the transcription events that lead to IL-18 generation rather than actual inflammasome regulation. The NLRP3 inflammasome has been shown to promote immune evasion in pancreatic cancer, specifically by differentiating T cells into pro-tumor populations (Th2, Th17, and T regs) and preventing the activation of tumor-killing CD8+ T cells (12). Our data suggest that NLRX1 can limit these immune evasion effects of the inflammasome by attenuating inflammasome signaling at the gene transcription level likely through the regulation of NF-κB, AKT, and/or MAPK signaling. Likewise, GM-CSF (*Csf2*) was significantly upregulated in Pan02^OE^ cells and downregulated in Pan02^KD^ cells. GM-CSF stimulates an anti-tumor immune response through priming CD4+ and CD8+ T cells, in part through recruiting and activating dendritic cells, and is currently being investigated for use in pancreatic cancer vaccines (4, 5, 58–60). Through upregulating *Csf2*, NLRX1 is potentially helpful in promoting immune recognition of pancreatic tumors.

In conclusion, we have characterized several phenotypes in murine Pan02 pancreatic tumor cells that are impacted by NLRX1. Our data suggest NLRX1 serves in a protective capacity against several cancer-associated biological processes in pancreatic cancer cells, including proliferation, evading cell death, migration, ROS signaling, and dysregulated mitochondrial function. The attenuation of these processes appears to be driven by a combination of suppressed NF-κB, MAPK, and AKT signaling. These outcomes suggest that further exploration of NLRX1 in pancreatic cancer, such as *in vivo* models of pancreatic cancer, is warranted. An orthotopic pancreatic tumor model would provide improved biological relevance to the data presented here, and the cell lines generated in the current study serve as an important tool for such future studies. Utilizing additional murine cell lines and human pancreatic cancer cell lines for future *in vitro* studies similar to those conducted here would also further enhance our understanding of NLRX1 in pancreatic cancer. The work presented here demonstrates that NLRX1 functions as a tumor suppressor in Pan02 cells and provides insight into mechanisms likely regulated by this unique pattern recognition receptor in the context of pancreatic cancer.

## Data availability statement

The original gene expression data presented in the study are publicly available. This data can be found here: https://www.ncbi.nlm.nih.gov/geo/query/acc.cgi?acc=GSE233493 (Accession Number: GSE233493).

## Author contributions

All authors participated in the drafting and writing of the manuscript. Project conceptualization and experimental design: MN-S, IA. Study execution and data analysis: MN-S, HM, MW, KL, KI, JT. Project supervision: MN-S, IA. Resources: IA. All authors contributed to the article and approved the submitted version.

## Supplementary material

Supplementary Figure 1 NLRX1 attenuates cancer-associated properties in Pan02 cells. **(A, B)**. Differences in proliferation as assessed by **(A)** automated counting and **(B)** MTT assay under TNF stimulation. n = 3-8 per cell line. **(C, D)**. Fluorometer measurements of MitoSOX staining for mitochondrial superoxide stimulated with **(C)** glucose as a positive control or **(D)** TNF. All data were analyzed using a two-way unpaired T test and shown as mean ± SE. *p ≤ 0.05, **p ≤ 0.01, ***p ≤ 0.001, ****p ≤ 0.0001.

Supplementary Figure 2 NLRX1 limits mitochondrial dysfunction and cellular energy production. **(A-D)**. From the Seahorse XF Cell Mito Stress kit, we observed multiple differences under TNF conditions in **(A)** spare respiratory capacity, **(B)** proton leak, **(C)** ATP production, and **(D)** glycolysis. n = 7 per cell line. All data were analyzed using a two-way unpaired T test and shown as mean ± SE. *p ≤ 0.05, **p ≤ 0.01, ***p ≤ 0.001, ****p ≤ 0.0001.

Supplementary Figure 3 Top 50 up- and down-regulated genes in unstimulated conditions. **(A-B)**. Based on the microarray transcriptomics assay, we list the top 50 up- and down-regulated DEGs between **(A)** Pan02^OE^ and Pan02^OE-CTL^ cells, and **(B)** between Pan02^KD^ and Pan02^KD-CTL^ cells in normal conditions. Listed in order of fold change.

Supplementary Figure 4 Top 50 up- and down-regulated genes in H_2_O_2_ conditions. **(A, B)**. Based on the microarray transcriptomics assay, we list the top 50 up- and down-regulated DEGs between **(A)** Pan02^OE^ and Pan02^OE-CTL^ cells, and **(B)** between Pan02^KD^ and Pan02^KD-CTL^ cells after low-dose H_2_O_2_ challenge. Listed in order of fold change.
