# Supplementary figures and images for: NLRX1 functions as a tumor suppressor in Pan02 pancreatic cancer cells
Source: Front Oncol. 2023 Jun 5;13:1155831. doi: 10.3389/fonc.2023.1155831 (PMC10277690; doi:10.3389/fonc.2023.1155831)

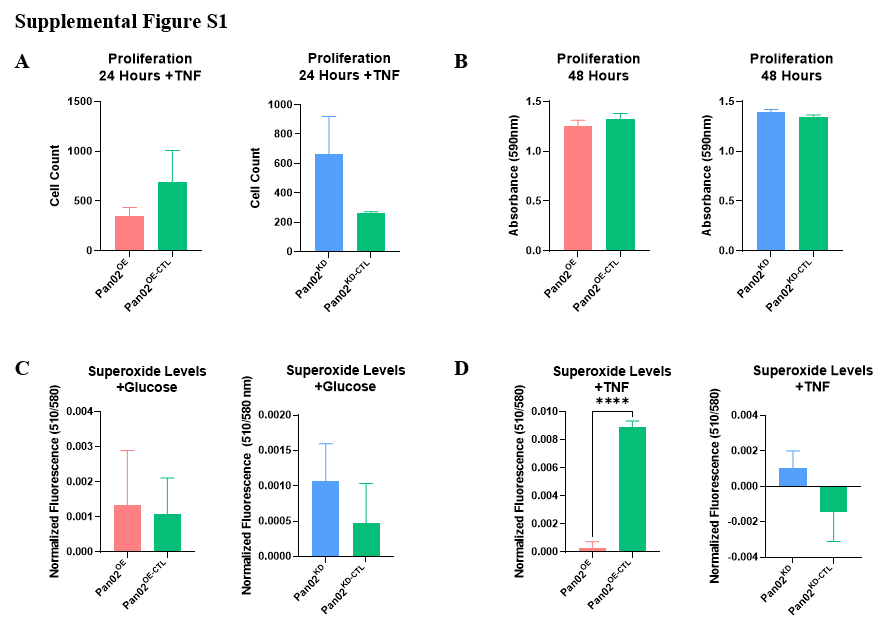

Supplement: Supplementary Figure 1 — NLRX1 attenuates cancer-associated properties in Pan02 cells. (A, B). Differences in proliferation as assessed by (A) automated counting and (B) MTT assay under TNF stimulation. n = 3-8 per cell line. (C, D). Fluorometer measurements of MitoSOX staining for mitochondrial superoxide stimulated with (C) glucose as a positive control or (D) TNF. All data were analyzed using a two-way unpaired T test and shown as mean ± SE. *p ≤ 0.05, **p ≤ 0.01, ***p ≤ 0.001, ****p ≤ 0.0001. [file Image_1.tif]

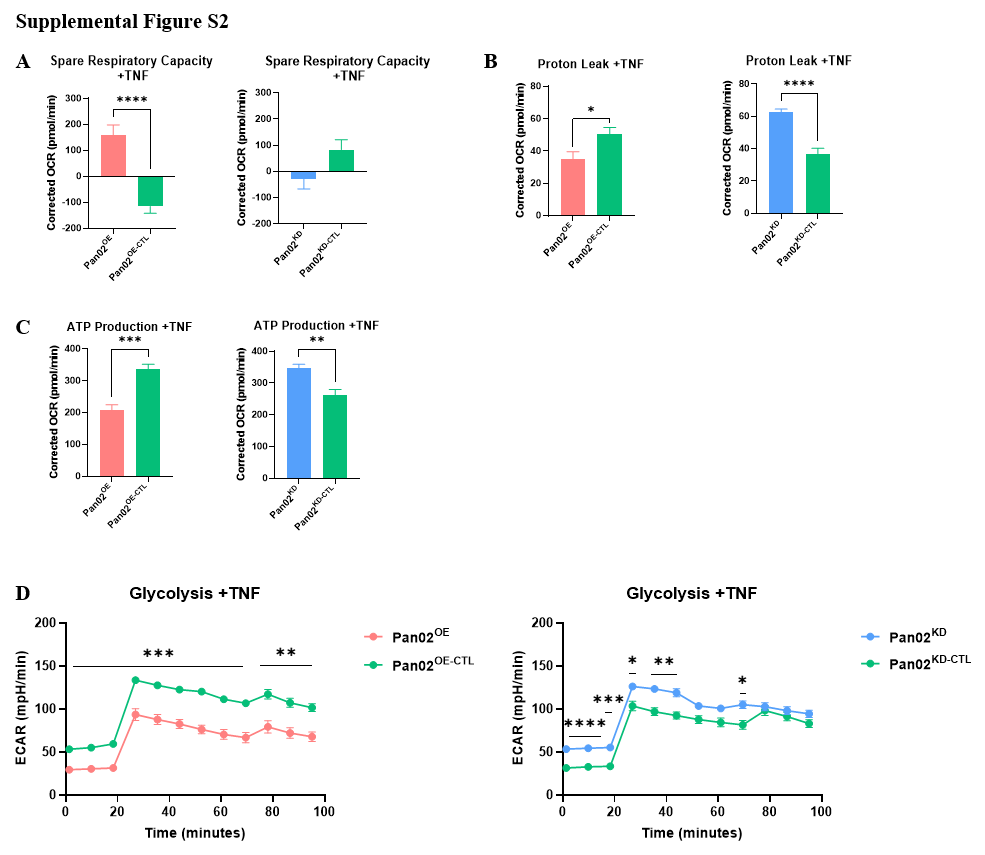

Supplement: Supplementary Figure 2 — NLRX1 limits mitochondrial dysfunction and cellular energy production. (A-D). From the Seahorse XF Cell Mito Stress kit, we observed multiple differences under TNF conditions in (A) spare respiratory capacity, (B) proton leak, (C) ATP production, and (D) glycolysis. n = 7 per cell line. All data were analyzed using a two-way unpaired T test and shown as mean ± SE. *p ≤ 0.05, **p ≤ 0.01, ***p ≤ 0.001, ****p ≤ 0.0001. [file Image_2.tif]

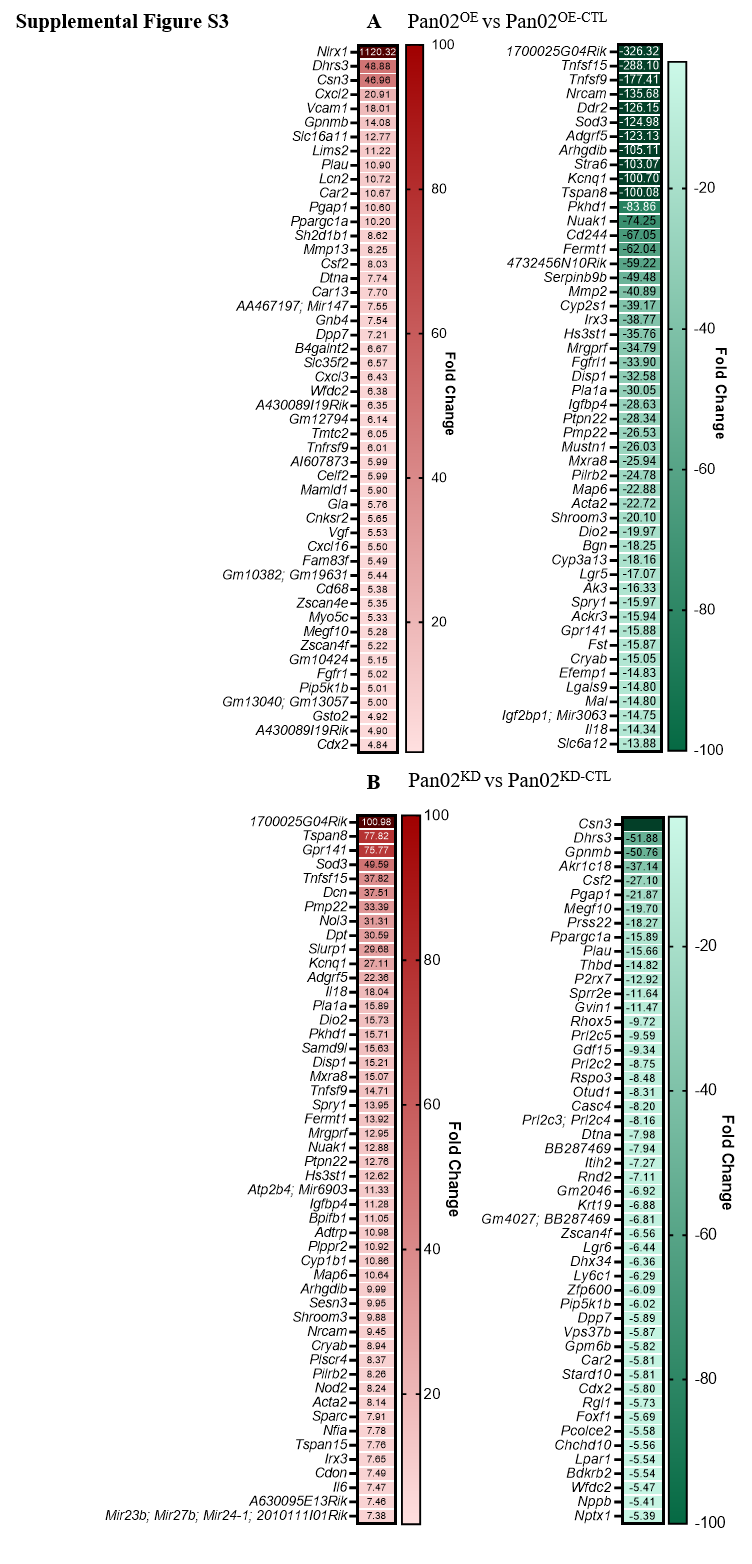

Supplement: Supplementary Figure 3 — Top 50 up- and down-regulated genes in unstimulated conditions. (A-B). Based on the microarray transcriptomics assay, we list the top 50 up- and down-regulated DEGs between (A) Pan02OE and Pan02OE-CTL cells, and (B) between Pan02KD and Pan02KD-CTL cells in normal conditions. Listed in order of fold change. [file Image_3.tif]

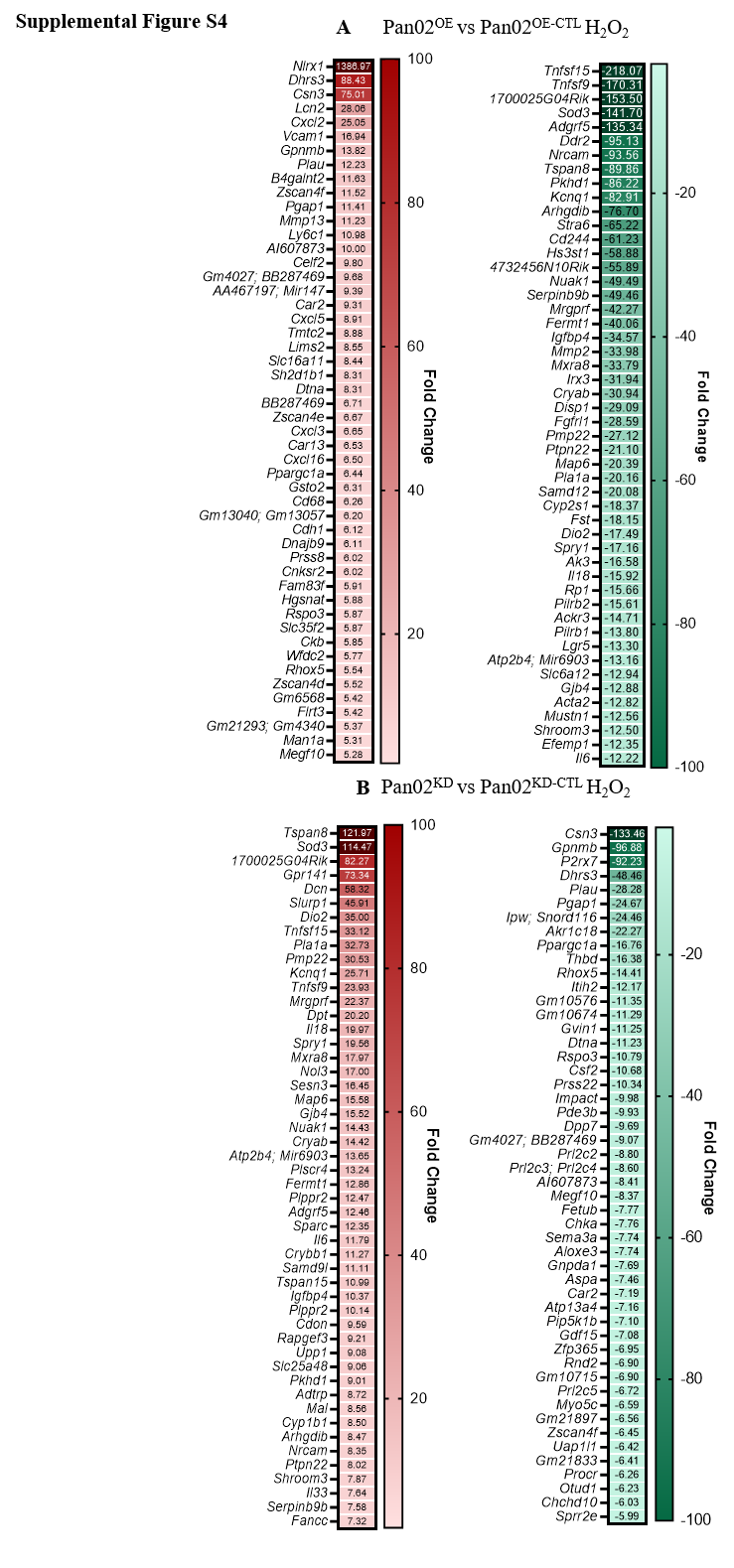

Supplement: Supplementary Figure 4 — Top 50 up- and down-regulated genes in H2O2 conditions. (A, B). Based on the microarray transcriptomics assay, we list the top 50 up- and down-regulated DEGs between (A) Pan02OE and Pan02OE-CTL cells, and (B) between Pan02KD and Pan02KD-CTL cells after low-dose H2O2 challenge. Listed in order of fold change. [file Image_4.tif]
